# Supplementary material for: A doubly stochastic renewal framework for partitioning spiking variability
Source: Nat Commun. 2025 Sep 30;16:8656. doi: 10.1038/s41467-025-63821-4 (PMC12484930; doi:10.1038/s41467-025-63821-4)
Supplement: Supplementary file 1 — Supplementary Information [file 41467_2025_63821_MOESM1_ESM.pdf]

Supplementary Information for: A Doubly Stochastic Renewal Framework for Partitioning Spiking Variability

Cina Aghamohammadi[1,2], Chandramouli Chandrasekaran[3,4,5,6], Tatiana A. Engel[1,2*]

[1]Princeton Neuroscience Institute, Princeton University, Princeton, NJ
[2]Cold Spring Harbor Laboratory, Cold Spring Harbor, NY, USA,
[3]Department of Anatomy & Neurobiology, Boston University, Boston, USA
[4]Department of Psychological and Brain Sciences, Boston University, Boston, USA
[5]Department of Biomedical Engineering, Boston University, Boston, MA
[6]Center for Systems Neuroscience, Boston University, Boston, MA
[*]Corresponding author e-mail: tatiana.engel@princeton.edu.

July 20, 2025

Contents

1 Supplementary Notes **1**
1.1 Derivations of mathematical results for renewal point processes 1
1.2 Robustness of estimation accuracy to bin size 8
1.3 Bias of the Deterministic Time Rescaling method 9
1.4 Biases of the Minimum Ratio method 12
1.5 Contribution of refractory period to spiking irregularity 14
1.6 Impact of dynamical regimes on spiking irregularity 15
1.7 Modulation of spiking irregularity and firing rate 16
1.8 Modeling serial ISI correlations with the DSR framework 19
1.9 Demšar comparison test for spiking irregularity ϕ across task conditions 20

1 Supplementary Notes

1.1 Derivations of mathematical results for renewal point processes

Definition 1 (Ordinary and equilibrium point processes). A point process can be called ordinary or equilibrium depending on the time at which we set the origin $t = 0$. The point process is called

ordinary if $t = 0$ is set at the time of the first spike. For an ordinary renewal point process, all interspike intervals (ISIs) are i.i.d. with the probability density $f(x)$. If an ordinary point process runs for a long time and then the time origin is picked randomly, then the resulting point process is called equilibrium [1]. This process is in a steady state because the time at which the process started is far from the time $t = 0$ at which we start sampling the process. For an equilibrium renewal point process, the interval from $t = 0$ to the first spike time t_1 has a different probability density $f_1(x)$ than all subsequent ISIs.

Theorem 1. Consider an equilibrium renewal point process defined by the ISI probability density $f(x)$, meaning that after generating a spike the probability that the next spike happens in the interval $[x, x + dx]$ is $f(x)dx$. Denoting the first three central moments of $f(x)$ by μ, σ^2, and μ_3, the mean $\mathrm{E}(\boldsymbol{N}_T)$ and the variance $\mathrm{Var}(\boldsymbol{N}_T)$ of the spike count $\boldsymbol{N}_T$ in a bin with the size T are

$$\begin{aligned} \mathrm{E}(\boldsymbol{N}_T) &= \frac{T}{\mu}, \\ \mathrm{Var}(\boldsymbol{N}_T) &= \frac{\sigma^2}{\mu^3}T + \frac{\sigma^4}{2\mu^4} + \frac{1}{6} - \frac{\mu_3}{3\mu^3} + \mathcal{O}(T^{-1}). \end{aligned}$$

Proof. Let us denote the time of the rth spike by x, its probability density function by $k_r(x)$, and its cumulative distribution function by $K_r(x)$. Let us define the functions:

$$\begin{aligned} H(T) &:= E(\boldsymbol{N}_T), \\ \psi(T) &:= E(\boldsymbol{N}_T(\boldsymbol{N}_T + 1)). \end{aligned}$$

With these definition, the variance of the spike count can be written as

$$\mathrm{Var}(\boldsymbol{N}_T) = \psi(T) - H(T) - H^2(T). \tag{1}$$

We next express $H(T)$ and $\psi(T)$ in terms of the density function $f(x)$ and its moments.

$$\begin{aligned} H(T) &= \sum_{r=0}^{\infty} r\mathbb{P}(\boldsymbol{N}_T = r) = \sum_{r=0}^{\infty} r(K_r(T) - K_{r+1}(T)) \\ &= \sum_{r=0}^{\infty} rK_r(T) - \sum_{r=0}^{\infty}(r+1)K_{r+1}(T) + \sum_{r=0}^{\infty} K_{r+1}(T) \\ &= \sum_{r=1}^{\infty} rK_r(T) - \sum_{r=1}^{\infty} rK_r(T) + \sum_{r=1}^{\infty} K_r(T) \\ &= \sum_{r=1}^{\infty} K_r(T). \end{aligned}$$

Similarly, we have

$$
\begin{aligned}
\psi(T) &= \sum_{r=0}^{\infty} r(r+1)\mathbb{P}(\boldsymbol{N}_T = r) = \sum_{r=1}^{\infty} r(r+1)(K_r(T) - K_{r+1}(T)) \\
&= \sum_{r=1}^{\infty} r(r+1)K_r(T) - \sum_{r=1}^{\infty}(r+1)(r+2)K_{r+1}(T) + \sum_{r=1}^{\infty} 2(r+1)K_{r+1}(T) \\
&= \sum_{r=1}^{\infty} r(r+1)K_r(T) - \sum_{r=2}^{\infty} r(r+1)K_r(T) + \sum_{r=2}^{\infty} 2rK_r(T) \\
&= 2K_1(T) + \sum_{r=2}^{\infty} 2rK_r(T) = \sum_{r=1}^{\infty} 2rK_r(T)\ .
\end{aligned}
$$

Using these two equalities, we write the Laplace transform of ψ and H, $\hat{\psi}(s) = \mathcal{L}\{\psi\}(s)$ and $\hat{H}(s) = \mathcal{L}\{H\}(s)$, in terms of the Laplace transform of k_r:

$$
\hat{H}(s) = \sum_{r=1}^{\infty} \hat{K}_r(s) = \sum_{r=1}^{\infty} \frac{1}{s}\hat{k}_r(s), \tag{2}
$$

$$
\hat{\psi}(s) = \sum_{r=1}^{\infty} 2r\hat{K}_r(s) = \sum_{r=1}^{\infty} \frac{2}{s} r\hat{k}_r(s). \tag{3}
$$

Let us denote the time of the first spike t_1, the interspike interval between the 1st and 2nd spikes by t_2, and similarly the time between $(r-1)^{\text{th}}$ and r^{th} spikes by t_r. Recalling that x is the time of the rth spike, we have $x = \sum_{i=1}^{r} t_i$. Thus,

$$
k_r(x) = (f_1 * \overbrace{f * \cdots * f}^{r-1})(x), \tag{4}
$$

where $f_1(.)$ is the probability density of the first interval t_1, and $f(.)$ is the probability density for the subsequent $r-1$ ISIs t_i. Since we have an equilibrium point process, f_1 and f are different functions. The operator $*$ denotes the time-domain convolution. Applying the Laplace transform to Eq. 4, we obtain

$$
\hat{k}_r(s) = \hat{f}_1(s)(\hat{f}(s))^{r-1}. \tag{5}
$$

It holds [1, 2]:

$$
\hat{f}_1(s) = \frac{1 - \hat{f}(s)}{\mu s}. \tag{6}
$$

Substituting Eq. 5 into Eqs. 2,3 and using Eq. 6, we obtain

$$
\hat{H}(s) = \frac{1}{\mu s^2}, \tag{7}
$$

$$
\hat{\psi}(s) = \frac{2}{\mu s^2(1 - \hat{f}(s))}. \tag{8}
$$

Taking the inverse Laplace transform in Eq. 7, we find

$$H(T) = \frac{T}{\mu}. \tag{9}$$

For a general case, we can not obtain a closed form for the inverse Laplace transform in Eq. 8 to calculate $\psi(T)$ exactly, but we can approximate it. If there exist $s_0 > 0$ such that for $\mathrm{Re}(s) > -s_0$, $\hat{f}(s)$ is analytical, then we can expand the Laplace transform $\hat{f}(s)$ in terms of moments m_n of the probability density $f(x)$ [3]:

$$\hat{f}(s) = \sum_{n=0}^{\infty} \frac{m_n}{n!}(-s)^n \approx 1 - \mu s + \frac{m_2}{2}s^2 - \frac{m_3}{3!}s^3.$$

Here $m_2 = \sigma^2 + \mu^2$ and $m_3 = \mu_3 + 3\mu\sigma^2 + \mu^3$ where $\mu_3 = E((x-\mu)^3)$. Now using Eq. 8, we can estimate $\hat{\psi}(s)$:

$$\hat{\psi}(s) \approx \frac{2}{\mu s^2(\mu s - \frac{m_2}{2}s^2 + \frac{m_3}{3!}s^3)} \; .$$

We expand this function around $s = 0$ and use $(1-x)^{-1} = 1 + x + x^2 + x^3 + \cdots$ to obtain

$$\begin{aligned}\hat{\psi}(s) &\approx \frac{2}{\mu^2 s^3}\frac{1}{(1 - \frac{m_2}{2\mu}s + \frac{m_3}{3!\mu}s^2)} \\ &\approx \frac{2}{\mu^2 s^3}\left(1 + \frac{m_2}{2\mu}s - \frac{m_3}{3!\mu}s^2 + \left(\frac{m_2}{2\mu}\right)^2 s^2 + \mathcal{O}(s^3)\right).\end{aligned}$$

Now we express m_2 and m_3 in terms of μ, σ and μ_3 and get

$$\begin{aligned}\hat{\psi}(s) &\approx \frac{2}{\mu^2 s^3}\left(1 + \frac{\sigma^2+\mu^2}{2\mu}s + \left(\left(\frac{\sigma^2+\mu^2}{2\mu}\right)^2 - \frac{\mu^3 + 3\mu\sigma^2 + \mu_3}{6\mu}\right)s^2 + \mathcal{O}(s^3)\right) \\ &= \frac{2}{\mu^2 s^3}\left(1 + \frac{\sigma^2+\mu^2}{2\mu}s + \left(\frac{\sigma^4}{4\mu^2} + \frac{\mu^2}{12} - \frac{\mu_3}{6\mu}\right)s^2 + \mathcal{O}(s^3)\right) \\ &= \frac{2}{\mu^2}\frac{1}{s^3} + \frac{\sigma^2+\mu^2}{\mu^3}\frac{1}{s^2} + \left(\frac{\sigma^4}{2\mu^4} + \frac{1}{6} - \frac{\mu_3}{3\mu^3}\right)\frac{1}{s} + \mathcal{O}(1)\; .\end{aligned}$$

Taking the inverse Laplace transform, we have

$$\psi(T) = \frac{1}{\mu^2}T^2 + \frac{\sigma^2+\mu^2}{\mu^3}T + \frac{\sigma^4}{2\mu^4} + \frac{1}{6} - \frac{\mu_3}{3\mu^3} + \mathcal{O}(T^{-1}). \tag{10}$$

Using Eqs. 1,9,10, we obtain the final result

$$\begin{aligned}\mathrm{Var}(\boldsymbol{N}_T) &= \frac{1}{\mu^2}T^2 + \frac{\sigma^2+\mu^2}{\mu^3}T + \frac{\sigma^4}{2\mu^4} + \frac{1}{6} - \frac{\mu_3}{3\mu^3} - \frac{1}{\mu}T - \frac{1}{\mu^2}T^2 + \mathcal{O}(T^{-1}) \\ &= \frac{\sigma^2}{\mu^3}T + \frac{\sigma^4}{2\mu^4} + \frac{1}{6} - \frac{\mu_3}{3\mu^3} + \mathcal{O}(T^{-1}).\end{aligned}$$

Theorem 2. For an equilibrium doubly stochastic renewal point process $\{g(\cdot), \boldsymbol{\lambda}\}$, where $\boldsymbol{\lambda}$ represents a stochastic firing rate assumed to be constant within a time bin T, the expected spike count $\mathrm{E}[\boldsymbol{N}_T|\boldsymbol{\lambda}]$ over a time bin T is given by $\boldsymbol{\lambda}T$.

Proof. Since $\boldsymbol{\lambda}$ is constant within a bin, then the spike generating process is a renewal point process fully defined by its ISI probability density, which we denote by $\boldsymbol{f_\lambda}$ since it depends on the value of $\boldsymbol{\lambda}$ in the bin. Theorem 1 asserts that for such a renewal process, $E[\boldsymbol{N}_T] = T/\mu$, where μ is first moment of the ISI probability density. Therefore, $\mathrm{E}[\boldsymbol{N}_T|\boldsymbol{\lambda}] = T/\boldsymbol{\mu}_{\boldsymbol{f_\lambda}}$, where $\boldsymbol{\mu}_{\boldsymbol{f_\lambda}}$ is the mean of the probability density $\boldsymbol{f_\lambda}$. Since $\boldsymbol{\lambda}$ is constant, by the definition of the renewal process, the density $\boldsymbol{f_\lambda}$ is a linear transformation of $g(\cdot)$. Therefore the mean of $\boldsymbol{f_\lambda}$ is

$$\boldsymbol{\mu}_{\boldsymbol{f_\lambda}} = \frac{\mu_g}{\boldsymbol{\lambda}} = \frac{1}{\boldsymbol{\lambda}}.$$

As a result, $\mathrm{E}[\boldsymbol{N}_T|\boldsymbol{\lambda}] = T/(1/\boldsymbol{\lambda}) = \boldsymbol{\lambda}T$.

Lemma 1. For an equilibrium inhomogeneous renewal point process $\{g(\cdot), \lambda(t)\}$ with firing rate $\lambda(t)$ that is a deterministic function of time fixed across realizations, the expected number of spikes $\boldsymbol{N}_T(t_0)$ within the bin $[t_0, t_0+T]$ is given by $\mathrm{E}[\boldsymbol{N}_T(t_0)] = T\overline{\lambda(t_0)}$, where

$$\overline{\lambda(t_0)} = \frac{1}{T}\int_{t_0}^{t_0+T} \lambda(t)dt$$

is the average firing rate over the bin.

Proof. Consider a small bin $[t, t+dt]$. Within this bin, the spike generating process is a renewal point process fully defined by its ISI probability density, which we denote by $f_{\lambda(t)}$ since it depends on the value of $\lambda(t)$ in the bin. Theorem 1 asserts that for such a renewal process, $\mathrm{E}[\boldsymbol{N}_{dt}(t)] = dt/\mu$, where μ is the first moment of the ISI probability density. Hence, $\mathrm{E}[\boldsymbol{N}_{dt}(t)] = dt/\mu_{f_{\lambda(t)}}$, where $\mu_{f_{\lambda(t)}}$ is the mean of the probability density $f_{\lambda(t)}$. By the definition of the renewal process, the density $f_{\lambda(t)}$ is a linear transformation of $g(\cdot)$. Therefore, the mean of $f_{\lambda(t)}$ is $\mu_{f_{\lambda(t)}} = \mu_g/\lambda(t) = 1/\lambda(t)$. As a result, $\mathrm{E}[\boldsymbol{N}_{dt}(t)] = \lambda(t)dt$, and consequently we have $\mathrm{E}[\boldsymbol{N}_T(t_0)] = \frac{1}{T}\int_{t_0}^{t_0+T} \lambda(t)dt$.

Theorem 3. For an equilibrium doubly stochastic renewal point process $\{g(\cdot), \boldsymbol{\lambda}(t)\}$ with a stationary stochastic firing rate $\boldsymbol{\lambda}(t)$ that varies across trials, the conditional expectation of the spike count $\boldsymbol{N}_T(t_0)$ in the interval $[t_0, t_0+T]$, conditioned on $\boldsymbol{\lambda}(t)$, is $\mathrm{E}[\boldsymbol{N}_T(t_0)|\boldsymbol{\lambda}(t)] = \int_{t_0}^{t_0+T} \boldsymbol{\lambda}(t)dt$.

Proof. From Lemma 1, for each realization $\lambda(t)$ of $\boldsymbol{\lambda}(t)$, we have $\mathrm{E}[\boldsymbol{N}_T(t_0)|\boldsymbol{\lambda}(t) = \lambda(t)] = \int_{t_0}^{t_0+T} \lambda(t)dt$. Therefore, $\mathrm{E}[\boldsymbol{N}_T(t_0)|\boldsymbol{\lambda}(t)] = \int_{t_0}^{t_0+T} \boldsymbol{\lambda}(t)dt$.

Corollary 1. If $\boldsymbol{\lambda}(t)$ is constant within the time bin T, taking the value $\boldsymbol{\lambda}(t) = \boldsymbol{\lambda}$ that varies across realizations, then $\mathrm{E}[\boldsymbol{N}_T(t_0)|\boldsymbol{\lambda}] = T\boldsymbol{\lambda}$.

Theorem 4. For an equilibrium doubly stochastic renewal point process $\{g(\cdot), \boldsymbol{\lambda}\}$ where $\boldsymbol{\lambda}$ is a stochastic firing rate that is constant within a time bin T varying across realizations, the following holds for the time bin T:

$$\mathrm{E}[\mathrm{Var}(\boldsymbol{N}_T|\boldsymbol{\lambda})] = \left(\frac{\sigma_g}{\mu_g}\right)^2 \mathrm{E}[\boldsymbol{N}_T] + \frac{1}{6} + \frac{1}{2}\left(\frac{\sigma_g}{\mu_g}\right)^4 - \frac{1}{3}\frac{\mu_{3g}}{\mu_g^3} + \mathcal{O}(T^{-1}).$$

Proof. Since $\boldsymbol{\lambda}$ is constant within a bin, the proof of Theorem 1 shows that we can expand $\mathrm{Var}(\boldsymbol{N}_T)$ in terms of powers of the bin size T where the powers go from $-\infty$ to 1. Since within a single bin, the spike generating process is a renewal point process defined by its ISI density, we can use the result of Theorem 1 to write

$$\mathrm{Var}(\boldsymbol{N}_T|\boldsymbol{\lambda}) = \sum_{i=-\infty}^{1} \boldsymbol{c_i} T^i.$$

Here $\boldsymbol{c_i}$ are random variables that depend on $\boldsymbol{f_\lambda}$.

For moderately large bin size $T \gg 1/\mathrm{E}[\boldsymbol{\lambda}]$, we can approximate $\mathrm{Var}(\boldsymbol{N}_T|\boldsymbol{\lambda})$ by the two largest powers of T. The proof of Theorem 1 shows that the coefficients $\boldsymbol{c_0}$ and $\boldsymbol{c_1}$ are functions of the mean, standard deviation and third central moment of the probability density $\boldsymbol{f_\lambda}$, which we denote by $\boldsymbol{\mu_{f_\lambda}}$, $\boldsymbol{\sigma_{f_\lambda}}$ and $\boldsymbol{\mu_{3f_\lambda}}$, respectively. Thus, using the result of Theorem 1, we can write

$$\mathrm{Var}(\boldsymbol{N}_T|\boldsymbol{\lambda}) = \frac{\boldsymbol{\sigma}^2_{\boldsymbol{f_\lambda}}}{\boldsymbol{\mu}^3_{\boldsymbol{f_\lambda}}} T + \frac{\boldsymbol{\sigma}^4_{\boldsymbol{f_\lambda}}}{2\boldsymbol{\mu}^4_{\boldsymbol{f_\lambda}}} + \frac{1}{6} - \frac{\boldsymbol{\mu}_{3\boldsymbol{f_\lambda}}}{3\boldsymbol{\mu}^3_{\boldsymbol{f_\lambda}}} + \mathcal{O}(T^{-1}).$$

By the statement of Theorem 2, we have $\mathrm{E}[\boldsymbol{N}_T|\boldsymbol{\lambda}] = \boldsymbol{\lambda} T = T/\boldsymbol{\mu_{f_\lambda}}$, from which we express T as $T = \boldsymbol{\mu_{f_\lambda}} \mathrm{E}[\boldsymbol{N}_T|\boldsymbol{\lambda}]$ and thus we rewrite the equation for $\mathrm{Var}(\boldsymbol{N}_T|\boldsymbol{\lambda})$:

$$\mathrm{Var}(\boldsymbol{N}_T|\boldsymbol{\lambda}) = \left(\frac{\boldsymbol{\sigma_{f_\lambda}}}{\boldsymbol{\mu_{f_\lambda}}}\right)^2 \mathrm{E}[\boldsymbol{N}_T|\boldsymbol{\lambda}] + \frac{1}{6} + \frac{1}{2}\left(\frac{\boldsymbol{\sigma_{f_\lambda}}}{\boldsymbol{\mu_{f_\lambda}}}\right)^4 - \frac{1}{3}\frac{\boldsymbol{\mu}_{3\boldsymbol{f_\lambda}}}{\boldsymbol{\mu}^3_{\boldsymbol{f_\lambda}}} + \mathcal{O}(T^{-1}).$$

Since $\boldsymbol{\lambda}$ is constant within a bin, by the definition of a renewal process, the density $\boldsymbol{f_\lambda}$ is a linear transformation of $g(\cdot)$, hence we can express the moments of $\boldsymbol{f_\lambda}$ in terms of the moments of $g(\cdot)$:

$$\boldsymbol{\mu_{f_\lambda}} = \frac{\mu_g}{\boldsymbol{\lambda}} = \frac{1}{\boldsymbol{\lambda}}, \quad \boldsymbol{\sigma}^2_{\boldsymbol{f_\lambda}} = \frac{\sigma_g^2}{\boldsymbol{\lambda}^2}, \quad \boldsymbol{\mu}_{3\boldsymbol{f_\lambda}} = \frac{\mu_{3g}}{\boldsymbol{\lambda}^3}.$$

In these relations, $\boldsymbol{\mu_{f_\lambda}}$, $\boldsymbol{\sigma_{f_\lambda}}$ and $\boldsymbol{\mu}_{3\boldsymbol{f_\lambda}}$ are random variables that fluctuate with $\boldsymbol{\lambda}$, which changes stochastically across time bins, whereas μ_g, σ_g and μ_{3g} are fixed because the function $g(\cdot)$ is fixed. Using these relations, we arrive at

$$\mathrm{Var}(\boldsymbol{N}_T|\boldsymbol{\lambda}) = \left(\frac{\sigma_g}{\mu_g}\right)^2 \mathrm{E}[\boldsymbol{N}_T|\boldsymbol{\lambda}] + \frac{1}{6} + \frac{1}{2}\left(\frac{\sigma_g}{\mu_g}\right)^4 - \frac{1}{3}\frac{\mu_{3g}}{\mu_g^3} + \mathcal{O}(T^{-1}).$$

For pair of random variables $\boldsymbol{X}$ and $\boldsymbol{Y}$, the Law of Total Expectation (LOTE) [4] states that $E(\boldsymbol{Y}) = \mathrm{E}[\mathrm{E}(\boldsymbol{Y}|\boldsymbol{X})]$. Taking the expectation over $\boldsymbol{\lambda}$ and applying LOTE in the previous equation, we obtain the final result

$$\mathrm{E}[\mathrm{Var}(\boldsymbol{N}_T|\boldsymbol{\lambda})] = \left(\frac{\sigma_g}{\mu_g}\right)^2 \mathrm{E}[\boldsymbol{N}_T] + \frac{1}{6} + \frac{1}{2}\left(\frac{\sigma_g}{\mu_g}\right)^4 - \frac{1}{3}\frac{\mu_{3g}}{\mu_g^3} + \mathcal{O}(T^{-1}).$$

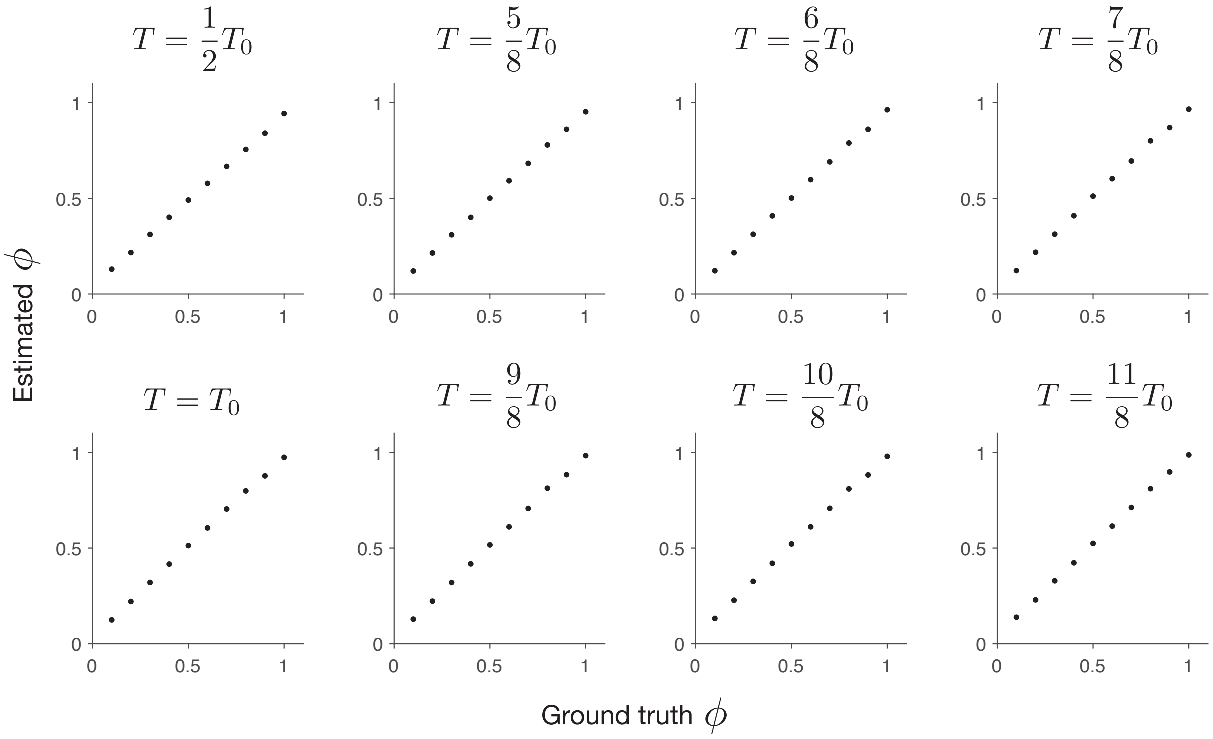

Supplementary Figure 1. Estimated ϕ versus ground-truth ϕ across bin sizes. We tested bin sizes ranging from $(1/2) \cdot T_0$ to $(11/8) \cdot T_0$, where $T_0 = 2/\mathrm{E}[\boldsymbol{\lambda}]$, using synthetic data generated from a doubly stochastic renewal process with a gamma renewal distribution and a drift-diffusion firing rate process (parameters as in Fig. 2). Each point represents the mean over 30 independent simulation runs.

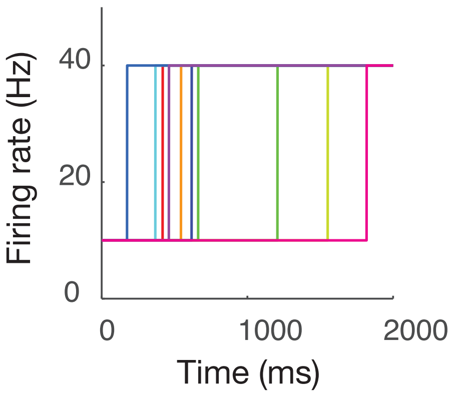

Supplementary Figure 2. Firing rate dynamics in the stepping model. The firing rate $\lambda(t)$ for each trial was generated using a stepping model, where $\lambda(t)$ began at 10 Hz and instantaneously transitioned to 40 Hz at a random time point within the trial duration. The jump times were sampled randomly across trials from a uniform distribution.

1.2 Robustness of estimation accuracy to bin size

We consider two criteria for selecting the bin size T used to estimate spiking irregularity. On one hand, we require $T > 1/\mathrm{E}[\boldsymbol{\lambda}]$ to ensure that the partitioning equation (Eq. 5 in the main text) holds. On the other hand, the bin size should be as small as possible, given the assumption that the firing rate remains constant within each bin. To satisfy both conditions, we set $T = 2/\mathrm{E}[\boldsymbol{\lambda}]$ for each neuron in experimental and synthetic data, where $\mathrm{E}[\boldsymbol{\lambda}]$ is the average firing rate of the neuron over the analysis period. Thus, the bin size depends on the neuron's average firing rate and varies across neurons. We performed two additional analyses to verify that our inference method is 1) robust and largely insensitive to bin size across a wide range of values, and 2) remains reliable even in the presence of rapid changes in firing rate.

First, we tested the robustness of our inference method to the specific choice of bin size. We used synthetic data generated from doubly stochastic renewal point processes with known ground-truth ϕ. Specifically, we chose $g(\cdot)$ to be a gamma distribution, and the firing rate $\lambda(t)$ on each trial sampled from a drift-diffusion process (as in lower row in Fig. 2 in the main text: trial duration = 2 s, number of trials = 100, upper threshold = 40 Hz, lower threshold = 10 Hz, D = 10 Hz^2/ms). We estimate ϕ with our method from synthetic data, with 30 independent runs for each value of ground truth ϕ. We denote the bin size used in the paper as $T_0 = 2/\mathrm{E}[\boldsymbol{\lambda}]$, and vary the bin size within the range from $(1/2) \cdot T_0$ to $(11/8) \cdot T_0$. We find that our estimation method remains highly accurate for this entire range of bin size (Supplementary Fig. 1).

Second, we tested the robustness of our method to rapid firing rate changes at certain times that are much faster than the chosen bin size $T = 2/\mathrm{E}[\boldsymbol{\lambda}]$. We used synthetic data generated from the doubly stochastic renewal point processes with known ground truth ϕ and the firing rate $\lambda(t)$ on each trial sampled from the stepping model: on each trial, the firing rate begins at 10 Hz and abruptly jumps to 40 Hz at a random time sampled from a uniform distribution (Supplementary Fig. 2). We generated synthetic data for 50 trials lasting 2 s. We estimated ϕ with our method from synthetic data, with 30 independent runs for each value of ground truth ϕ. We vary the bin

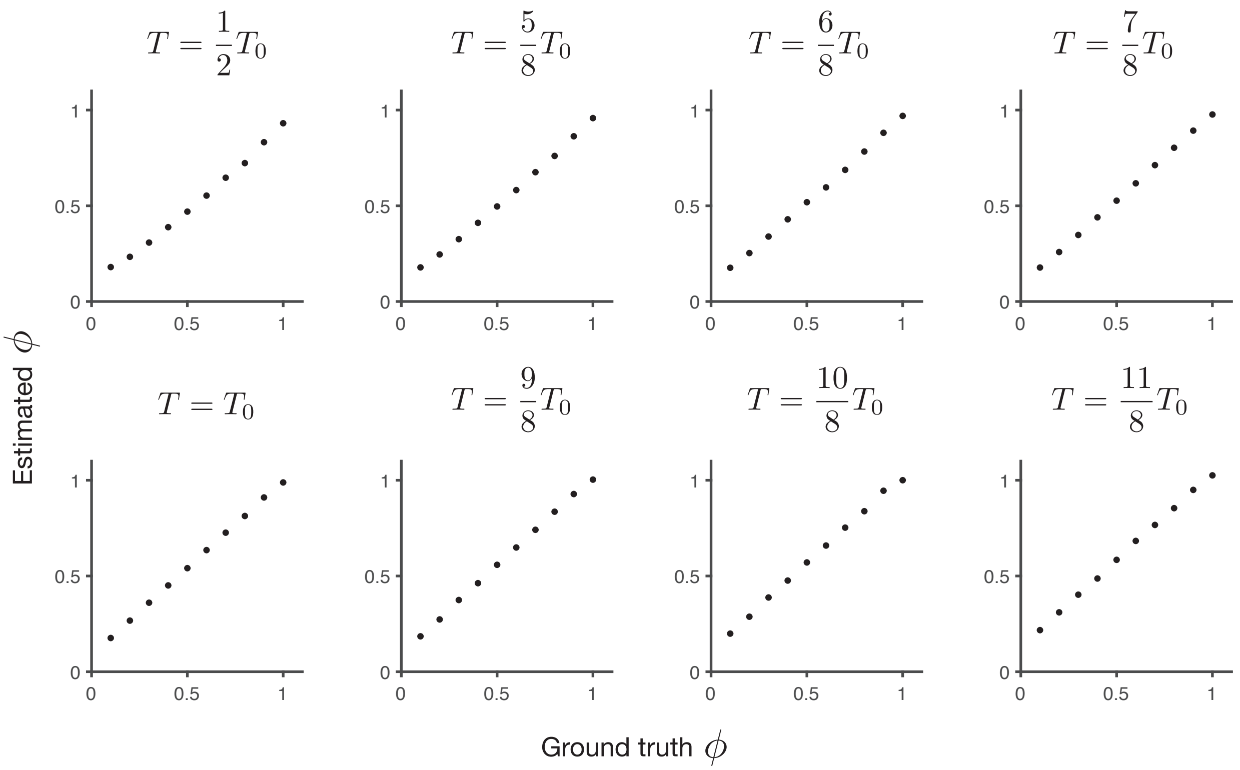

Supplementary Figure 3. Estimated ϕ versus ground-truth ϕ for synthetic data with occasional rapid changes in firing rate. We tested bin sizes ranging from $(1/2) \cdot T_0$ to $(11/8) \cdot T_0$, where $T_0 = 2/\mathrm{E}[\boldsymbol{\lambda}]$, using synthetic data generated from a doubly stochastic renewal process with a gamma renewal distribution and a stepping firing rate process (parameters as in Supplementary Fig. 2). Each point represents the mean over 30 independent simulation runs.

size within the range from from $(1/2) \cdot T_0$ to $(11/8) \cdot T_0$. We find that our estimation method remains accurate in this scenario where the firing rate exhibits occasional rapid changes in each trial (Supplementary Fig. 3).

Intuitively, the estimation method remains robust if rapid changes in the firing rate are relatively rare. Suppose each trial lasts 2 s and the average firing rate is approximately 20 Hz. We then choose a bin size of 100 ms, resulting in 20 bins per trial. If the firing rate jumps only once per trial, about 5% of bins include a jump in firing rate. Thus, across 20 trials, rapid firing-rate changes occur in only one or two trials on average, with negligible impact on the estimation accuracy. In contrast, if firing rate changes rapidly in most bins, our inference method would attribute such ongoing rapid changes to spiking irregularity.

1.3 Bias of the Deterministic Time Rescaling method

To estimate the bias of the Deterministic Time Rescaling (DTR) method, we consider a doubly stochastic renewal point process $\{g(\cdot), \boldsymbol{\lambda}(t)\}$ in a particular time bin $[t, t+T]$. We assume that the firing rate is constant within a single bin but fluctuates from trial to trial with a uniform

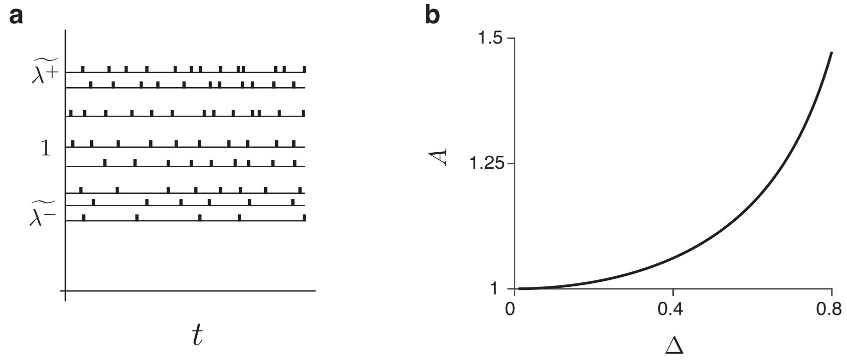

Supplementary Figure 4. Estimation error of the deterministic time rescaling method. **a**, We consider the case in which the firing rate is constant over time within a trial and fluctuates from trial to trial sampled from a uniform distribution over the interval $[\lambda^-, \lambda^+]$ Hz. Since the DTR method makes the assumption that the variance of the firing rate is zero, it mixes the ISIs from different trials with different firing rates. Intuitively, this mixing increases the estimated variance of ISIs. **b**, The estimated ϕ_{DTR} is linearly related to the ground-truth ϕ via $\phi_{\mathrm{DTR}} = A\phi + (A-1)$. The parameter A is a function of $\Delta = (\lambda^+ - \lambda^-)/(2\lambda_0)$ which controls the trial-to-trial variance of the firing rate.

distribution over the interval $[\lambda^-, \lambda^+]$.

The DTR method assumes that the firing rate is deterministic and estimates the firing rate simply as the average spike count in a bin across trials. In our setting, this estimate produces

$$\lambda_0 := \frac{1}{T}\mathrm{E}(N) = \frac{1}{T}\mathrm{E}(\mathrm{E}(N|\lambda)) = \frac{1}{T}\mathrm{E}(\lambda T) = \mathrm{E}(\lambda) = \frac{\lambda^- + \lambda^+}{2}.$$

Using this estimated firing rate λ_0, the DTR method then rescales ISIs to map the spikes from real to operational time. This rescaling maps the highest firing rate λ^+ to $\widetilde{\lambda^+} = \lambda^+/\lambda_0 > 1$, and the lowest firing rate λ^- to $\widetilde{\lambda^-} = \lambda^-/\lambda_0 < 1$, and λ_0 maps to $\widetilde{\lambda_0} = 1$ Hz. Finally, the DTR method estimates ϕ as CV^2 of the rescaled ISIs by estimating the mean μ_n and standard deviation σ_n of the rescaled ISIs. To calculate what this estimate will produce, we need to calculate the probability density $f(\tau)$ of rescaled ISIs. Rescaling time with the DTR method mixes ISIs from different trials, assuming they all came from the same firing rate, into a single $f(\tau)$ (Supplementary Fig. 4a). Thus, we have

$$f(\tau) = \int_{\widetilde{\lambda^-}}^{\widetilde{\lambda^+}} f(\tau|\lambda)\mathbb{P}(\lambda)d\lambda.$$

Since within a bin, the only contribution of λ is to scale the ISIs, the conditional distribution is $f(\tau|\lambda) = \lambda g(\lambda\tau)$. Since we assumed a uniform distribution for the firing rate across trials, $\mathbb{P}(\lambda) = \lambda/(\widetilde{\lambda^+} - \widetilde{\lambda^-})$ which leads to

$$f(\tau) = \frac{1}{\widetilde{\lambda^+} - \widetilde{\lambda^-}} \cdot \int_{\widetilde{\lambda^-}}^{\widetilde{\lambda^+}} \lambda g(\lambda\tau)d\lambda.$$

In theory, the first and second moments of the ISI probability density $g(\cdot)$ are

$$\begin{aligned}\mu_0 &= \int_0^\infty \tau g(\tau) d\tau = 1,\\ \sigma_0^2 &= \int_0^\infty (\tau-\mu_0)^2 g(\tau) d\tau = \phi.\end{aligned}$$

Using the DTR method, then mean μ_n of the rescaled ISIs is

$$\begin{aligned}\mu_n &= \int_0^\infty \tau f(\tau) d\tau = \int_0^\infty d\tau\tau \int_{\widetilde{\lambda^-}}^{\widetilde{\lambda^+}} f(\tau|\lambda)\mathbb{P}(\lambda) d\lambda\\ &= \frac{1}{\widetilde{\lambda^+}-\widetilde{\lambda^-}} \int_0^\infty d\tau\tau \int_{\widetilde{\lambda^-}}^{\widetilde{\lambda^+}} \lambda g(\lambda\tau) d\lambda = \frac{1}{\widetilde{\lambda^+}-\widetilde{\lambda^-}} \int_{\widetilde{\lambda^-}}^{\widetilde{\lambda^+}} d\lambda\lambda \int_0^\infty \tau g(\lambda\tau) d\tau\\ &= \frac{1}{\widetilde{\lambda^+}-\widetilde{\lambda^-}} \int_{\widetilde{\lambda^-}}^{\widetilde{\lambda^+}} d\lambda\lambda \int_0^\infty \frac{\tau'}{\lambda} g(\tau')\frac{d\tau'}{\lambda} = \frac{1}{\widetilde{\lambda^+}-\widetilde{\lambda^-}} \int_{\widetilde{\lambda^-}}^{\widetilde{\lambda^+}} \frac{1}{\lambda} d\lambda \int_0^\infty \tau' g(\tau') d\tau' = \frac{\ln(\widetilde{\lambda^+}/\widetilde{\lambda^-})}{\widetilde{\lambda^+}-\widetilde{\lambda^-}}.\end{aligned}$$

We see that the estimated mean ISI μ_n is not equal 1 s despite the fact that the linear average of the firing rate is $\widetilde{\lambda}_0 = 1$ Hz. This result shows that neglecting fluctuations in the firing rate and replacing it with a deterministic firing rate will produce the estimated mean ISI that is not necessarily consistent with the average firing rate. If trial-to-trial fluctuations of the firing rate are small compared to the average firing rate: $\widetilde{\lambda^+}-\widetilde{\lambda^-} \ll \widetilde{\lambda}_0 = 1$, then we recover $\mu_n \approx 1$ s. The equality only holds in the limit of zero variance, that is, for a deterministic firing rate.

For the standard deviation of the rescaled ISIs σ_n, we have

$$\begin{aligned}\widetilde{\sigma}_n^2 &= \int_0^\infty (\tau-\widetilde{\mu}_n)^2 f(\tau) d\tau = \int_0^\infty (\tau-\widetilde{\mu}_n)^2 \int_{\widetilde{\lambda^-}}^{\widetilde{\lambda^+}} f(\tau|\lambda)\mathbb{P}(\lambda) d\lambda d\tau\\ &= \frac{1}{\widetilde{\lambda^+}-\widetilde{\lambda^-}} \int_{\widetilde{\lambda^-}}^{\widetilde{\lambda^+}} d\lambda\lambda \int_0^\infty (\tau-\widetilde{\mu}_n)^2 g(\lambda\tau) d\tau\\ &= \frac{1}{\widetilde{\lambda^+}-\widetilde{\lambda^-}} \int_{\widetilde{\lambda^-}}^{\widetilde{\lambda^+}} d\lambda\lambda \int_0^\infty \left(\frac{\tau'}{\lambda}-\widetilde{\mu}_n\right)^2 g(\tau')\frac{d\tau'}{\lambda}\\ &= \frac{1}{\widetilde{\lambda^+}-\widetilde{\lambda^-}} \int_{\widetilde{\lambda^-}}^{\widetilde{\lambda^+}} d\lambda \int_0^\infty \left(\frac{\tau'^2}{\lambda^2}+\widetilde{\mu}_n^2-2\frac{\tau'}{\lambda}\widetilde{\mu}_n\right) g(\tau') d\tau'\\ &= \frac{1}{\widetilde{\lambda^+}-\widetilde{\lambda^-}} \int_{\widetilde{\lambda^-}}^{\widetilde{\lambda^+}} \left(\frac{1+\phi}{\lambda^2}+\widetilde{\mu}_n^2-2\frac{\widetilde{\mu}_n}{\lambda}\right) d\lambda\\ &= \frac{1}{\widetilde{\lambda^+}-\widetilde{\lambda^-}} \left((1+\phi)\left(\frac{1}{\widetilde{\lambda^-}}-\frac{1}{\widetilde{\lambda^+}}\right)+\widetilde{\mu}_n^2(\widetilde{\lambda^+}-\widetilde{\lambda^-})-2\widetilde{\mu}_n \ln\left(\frac{\widetilde{\lambda^+}}{\widetilde{\lambda^-}}\right)\right)\\ &= \frac{(1+\phi)}{\widetilde{\lambda^+}\widetilde{\lambda^-}}+\widetilde{\mu}_n^2-2\widetilde{\mu}_n^2 = \frac{(1+\phi)}{\widetilde{\lambda^+}\widetilde{\lambda^-}}-\widetilde{\mu}_n^2\ .\end{aligned}$$

Then the estimated ϕ_{DTR} is

$$\phi_{\text{DTR}} = \frac{\widetilde{\sigma}_n^2}{\widetilde{\mu}_n^2} = A\phi + (A-1),$$

where

$$A = \frac{(\widetilde{\lambda^+} - \widetilde{\lambda^-})^2}{\widetilde{\lambda^+}\widetilde{\lambda^-}(\ln(\widetilde{\lambda^+}/\widetilde{\lambda^-}))^2} = \frac{4\Delta^2}{(1-\Delta^2)(\ln(\frac{1+\Delta}{1-\Delta}))^2}.$$

Here we defined $\Delta = \frac{\lambda^+ - \lambda^-}{2\lambda_0} = \frac{\lambda^+ - \lambda^-}{\lambda^+ + \lambda^-}$, which is a control parameter that indicates how much the firing rate process deviates from the deterministic assumption. $\Delta = 0$ corresponds to the deterministic firing rate which leads to $A = 1$ and we recover $\phi_{\mathrm{DTR}} = \phi$. The dependence of A on Δ is a monotonically increasing function (Supplementary Fig. 4b), hence increasing Δ leads to a larger error in ϕ_{DTR}.

1.4 Biases of the Minimum Ratio method

The Minimum Ratio (MR) method for partitioning spiking variability [5] starts with an assumption

$$\mathrm{E}[\mathrm{Var}(\boldsymbol{N}_T|\boldsymbol{\lambda})] = \phi\mathrm{E}[\boldsymbol{N}_T], \tag{11}$$

which is suggested to be based on the renewal theory, although the original paper does not specify the underlying assumptions about the spike generating process [5]. To see whether this equation might be consistent with the renewal theory, let us consider a renewal point process $\{g(\cdot), \lambda\}$ with a deterministic firing rate (constant in time and across trials), for which it holds [1, 2]

$$\lim_{T\to\infty} \frac{\mathrm{Var}(\boldsymbol{N}_T)}{\mathrm{E}[\boldsymbol{N}_T]} = \text{constant}.$$

However, this relation holds only in the limit of an infinite bin size $T \to \infty$ and for a constant firing rate. Before we can evaluate the relationship between $\mathrm{E}[\mathrm{Var}(\boldsymbol{N}_T|\boldsymbol{\lambda})]$ and $\mathrm{E}[\boldsymbol{N}_T]$ for a doubly stochastic renewal point process, we first need to define what this process is, that is, specify the spike generating process because in the absence of a mathematical model, the partitioning of variability is ambiguous [6]. Using our definition of a generative model for a doubly stochastic renewal point process, we derived the expression for $\mathrm{E}[\mathrm{Var}(\boldsymbol{N}_T|\boldsymbol{\lambda})]$ (Eq. 12 in Methods, Theorem 3), thus we can compute

$$\lim_{T\to\infty} \frac{\mathrm{E}[\mathrm{Var}(\boldsymbol{N}_T|\boldsymbol{\lambda})]}{\mathrm{E}[\boldsymbol{N}_T]} = \lim_{T\to\infty} \frac{\phi\mathrm{E}[\boldsymbol{N}_T] + \frac{1}{6} + \frac{1}{2}\phi^2 - \frac{1}{3}\psi(\phi) + \mathcal{O}(T^{-1})}{\mathrm{E}[\boldsymbol{N}_T]} = \phi.$$

Thus, the suggested relation Eq. 11 holds for our definition of a doubly stochastic point process, but also only for an infinite bin size in the limit $T \to \infty$. For a finite bin size, this ratio generally depends on the bin size because the expected spike count $\mathrm{E}[\boldsymbol{N}_T]$ increases with T. Therefore, the assumption Eq. 11 in the MR method is not usually true. It is valid for any bin size only in a special case when $\psi(\phi) = \frac{3}{2}\phi^2 + \frac{1}{2}$ which does not generally hold. For example, if the ISI probability density $g(\cdot)$ is the gamma distribution, then $\psi(\phi) = 2\phi^2$ (Methods), from which we see that this condition only holds when $\phi = 1$, that is, for the Poisson process.

Using the assumption Eq. 11, the MR method partitions the variability as

$$\mathrm{Var}(\mathrm{E}[\boldsymbol{N}_T|\boldsymbol{\lambda}]) = \mathrm{Var}(\boldsymbol{N}_T) - \phi\mathrm{E}[\boldsymbol{N}_T].$$

Since variance must be non-negative ($\mathrm{Var}(\mathrm{E}[\boldsymbol{N}_T|\boldsymbol{\lambda}]) \geqslant 0$), the MR method estimates ϕ by calculating the minimum Fano factor estimated from data for all time bins:

$$\phi \approx \phi_{\mathrm{MR}} = \min_t \left\{ \frac{\mathrm{Var}(\boldsymbol{N}_T(t))}{\mathrm{E}[\boldsymbol{N}_T(t)]} \right\}. \tag{12}$$

This MR method for estimating ϕ contains multiple sources of bias. First, assuming the firing rate $\boldsymbol{\lambda}(t)$ changes smoothly on a timescale longer than the bin size T, we derived the correct partitioning equation for doubly stochastic renewal point processes (Eq. 12 in Methods, Theorem 3):

$$\mathrm{Var}(\mathrm{E}[\boldsymbol{N}_T|\boldsymbol{\lambda}]) = \mathrm{Var}(\boldsymbol{N}_T(t)) - \phi \mathrm{E}[\boldsymbol{N}_T(t)] - \frac{1}{6} - \frac{1}{2}\phi^2 + \frac{1}{3}\psi(\phi) + \mathcal{O}(T^{-1}).$$

Using the fact that $\mathrm{E}[\boldsymbol{N}_T(t)] = TE[\boldsymbol{\lambda}(t)]$ (Theorem 2) and $\mathrm{Var}(\boldsymbol{\lambda}(t)T) = T^2\mathrm{Var}(\boldsymbol{\lambda}(t))$, we can approximate the ratio of $\mathrm{Var}(\boldsymbol{N}_T(t))$ to $\mathrm{E}[\boldsymbol{N}_T(t)]$ for a finite bin size as:

$$\frac{\mathrm{Var}(\boldsymbol{N}_T(t))}{\mathrm{E}[\boldsymbol{N}_T(t)]} \approx \phi + T \cdot \frac{\mathrm{Var}(\boldsymbol{\lambda}(t))}{\mathrm{E}[\boldsymbol{\lambda}(t)]} + \frac{1}{T} \cdot \frac{\frac{1}{6} + \frac{1}{2}\phi^2 - \frac{1}{3}\psi(\phi)}{\mathrm{E}[\boldsymbol{\lambda}(t)]}.$$

From this equation and the definition of ϕ_{MR} Eq. 12, we obtain the relation between the ground truth ϕ and ϕ_{MR} estimated with the MR method:

$$\phi_{\mathrm{MR}} \approx \min_t \left\{ \phi + T \cdot \frac{\mathrm{Var}(\boldsymbol{\lambda}(t))}{\mathrm{E}[\boldsymbol{\lambda}(t)]} + \frac{1}{T} \cdot \frac{\frac{1}{6} + \frac{1}{2}\phi^2 - \frac{1}{3}\psi(\phi)}{\mathrm{E}[\boldsymbol{\lambda}(t)]} \right\}.$$

This equation shows that the MR method has several sources of bias. The first source is the term

$$T \cdot \frac{\mathrm{Var}(\boldsymbol{\lambda}(t))}{\mathrm{E}[\boldsymbol{\lambda}(t)]},$$

which is proportional to the trial-to-trial variance of the firing rate and linearly grows with the bin size T. This source of bias leads to an overestimation of ϕ and vanishes only when the variance of the firing rate is zero. The second source is the term

$$\frac{1}{T} \cdot \frac{\frac{1}{6} + \frac{1}{2}\phi^2 - \frac{1}{3}\psi(\phi)}{\mathrm{E}[\boldsymbol{\lambda}(t)]}.$$

We can predict the sign of this term in the case when $g(\cdot)$ is a gamma distribution. The sign is positive leading to an overestimation of ϕ if the ground-truth $\phi < 1$ (sub-Poisson spiking irregularity). The sign is negative leading to underestimation of ϕ if the ground-truth $\phi > 1$ (super-Poisson spiking irregularity). This source of bias decreases with increasing bin size and average firing rate. The third source of bias arises from the $\min_t\{\cdot\}$ operator. The term inside this operator is estimated for every bin from a finite number of trials in the data. Due to the estimation error, the estimated term in every bin comes from a sampling distribution with a finite width, which goes to zero as the number of trails goes to infinity. Assuming ϕ is constant across time bins [5], these estimates are samples drawn from the same distribution and the number of samples is equal to the number of time bins. Instead of reducing estimation noise by averaging

samples, the operator $\min_t\{\cdot\}$ always chooses the minimum across samples, which always leads to underestimation of ϕ, and this bias does not decrease with the number of samples.

Mixing these three sources of bias leads to unpredictable errors in ϕ_{MR} estimated with the MR method. ϕ_{MR} estimated by this method has been used to compute the variance of the firing rate $\mathrm{Var}(\boldsymbol{\lambda}(t))$ using Eq. 12 [5], in which case the error in estimating ϕ propagates to the estimate of the firing-rate variance and how it depends on time. For example, if the mean firing rate increases with time, an underestimation of ϕ creates an increasing trend in $\mathrm{Var}(\boldsymbol{\lambda}(t))$, even if the actual firing-rate variance is constant in time. Since $\mathrm{Var}(\boldsymbol{\lambda}(t))$ is widely used as a metric to distinguish different classes of models for neural computation [5, 7, 8], unpredictable errors in the computation can lead to unreliable results and inaccurate interpretation of the data.

1.5 Contribution of refractory period to spiking irregularity

After firing a spike, neurons enter a refractory period during which their ability to generate another spike is diminished for a duration ranging from approximately 4 to 12 ms [9]. To sustain a high firing rate, the interspike intervals (ISIs) must be short, and when ISIs become sufficiently brief, their duration is constrained by the refractory period. Therefore, the refractory period can contribute to spiking irregularity.

We performed two analyses to verify that the refractory period is not the sole source of the negative correlation between spiking irregularity and firing rate that we observed in the experimental data (Fig. 4). First, we estimated the mean firing rate above which the refractory period significantly affects spiking irregularity. Second, we systematically removed high firing-rate neurons from the experimental data to confirm that the negative correlation between the firing rate and spiking irregularity is not solely driven by spiking regularity of high firing-rate neurons.

First, we estimated the firing rate above which the refractory period significantly impacts spiking irregularity. We considered an integrate-and-fire type neuron that fires a spike when the voltage reaches a threshold V_{th}, upon which the voltage is reset to V_r after a fixed refractory period RP. The neuron receives a presynaptic input spike train following a homogeneous renewal process with a constant firing rate λ_{in}. Each presynaptic spike increments the membrane potential by an amount J. In the stationary regime, we can decompose the postsynaptic interspike interval (ISI) into the refractory period and a random component: $ISI = RP + X$. The probability distribution of random variable X generally depends on parameters such as neuron properties, input statistics, and RP. Let us assume that $(V_{\mathrm{th}} - V_r)/J \gg 1$, meaning that the neuron needs to receive $n \gg 1$ presynaptic spikes to generate the next spike after reset. We can then write $X = t_0 + \sum_{i=1}^{n-1} t_i$, where t_0 is the time between the end of RP and the arrival of the first presynaptic spike, and t_i is the ISI between the i-th and $(i+1)$-th presynaptic spikes. Since the input process is renewal, only t_0 term in X is influenced by the refractory period, and X is approximately independent of RP for large n. For constant input firing rate, we can compute the spiking irregularity using ISI moments in real time, instead of operational time, to obtain

$$\phi = \frac{\sigma_{ISI}^2}{\mu_{ISI}^2} = \frac{\sigma_X^2}{(RP + \mu_X)^2} = \frac{CV_X^2}{(\frac{RP}{\mu_X} + 1)^2}. \tag{13}$$

Here CV_X is the coefficient of variation of X, and the average firing rate of the neuron is $fr = (RP + X)^{-1}$. This equation shows that spiking irregularity decreases with increasing RP. In the limit $\mu_X \gg RP$ (equivalently, $RP \ll 1/fr$), the refractory period has negligible effect and

$\phi \approx {CV_X}^2$. In the opposite limit $\mu_X \ll RP$ (equivalently, $RP \approx 1/fr$), the refractory period dominates and $\phi \approx 0$. In between these two extremes, both RP and X jointly determine the spiking irregularity. The refractory period (including the duration of the action potential) of neurons typically ranges from approximately 4 to 12 ms [9]. Hence, the firing rate above which the refractory period dominates spiking irregularity ranges from 1/12 ms ≈ 83 Hz to 1/4 ms≈ 250 Hz.

Second, we examined the potential effect of the refractory period on the negative correlation between the firing rate and spiking irregularity in experimental data. The correlation remained significant when restricting analysis to neurons with firing rates below 83 Hz (LIP: $r = 0.51$, $p = 0.0001$; V4: $r = 0.24$, $p = 0.0001$) and below 50 Hz (LIP: $r = 0.48$, $p = 0.0015$; V4: $r = 0.22$, $p = 0.0002$). Therefore, the negative correlation between firing rate and spiking irregularity does not arise solely from the refractory period effects of high firing-rate neurons.

1.6 Impact of dynamical regimes on spiking irregularity

We used a random, sparsely connected balanced spiking network model [10] to investigate how recurrent dynamics give rise to diverse spiking irregularity across neurons. In this model, each neuron receives exactly the same number of excitatory and inhibitory connections, allowing us to test a separate mechanism for generating heterogeneous spiking irregularity, which does not rely on differences in synaptic input balance (Fig. 6). In addition, this model generates variability solely from its internal recurrent dynamics, whereas the three-layer spatial network model (Fig. 6) includes a Poisson layer as an external source of variability.

We simulated a random balanced network model consisting of $N = 10,000$ leaky integrate-and-fire neurons. A fraction $f = 0.8$ of these neurons are excitatory, and the remaining neurons are inhibitory. The membrane potential dynamics of the ith neuron are given by

$$\tau_m \frac{dV_i}{dt} = -V_i + \mu_0 + RI_i(t), \tag{14}$$

where

$$RI_i(t) = \tau_m \sum_j J_{ij} \sum_k \delta(t - t_k^j - \Delta). \tag{15}$$

Here t_k^j is the kth presynaptic spike from neuron j and $\tau_m = 20$ ms, $\Delta = 0.55$ ms, $\mu_0 = 24$ mV. When the membrane potential crosses the threshold $V_{\text{th}} = 20$ mV, an action potential is emitted and the membrane potential is reset to the $V_r = 10$ mV. All neurons have the same refractory period of $\tau_{\text{ref}} = 0.5$ ms. Each neuron receives the same number $C = 1,000$ incoming connections. The fraction f of these connections are excitatory, and the remaining connections are inhibitory. All excitatory synapses have the same strength J, and all inhibitory synapses have the same strength gJ, with $g = 5$.

This random, sparsely connected balanced spiking network model exhibits two distinct types of asynchronous activity [10], each with different computational implications. For weak synaptic coupling, the network operates in the classical asynchronous regime, characterized by unstructured activity with similarly irregular firing across all neurons (Supplementary Fig. 5a). In this regime, external input produces a highly redundant response across neurons, facilitating information transmission. For strong synaptic couplings, the network operates in a heterogeneous asynchronous regime characterized by rich internal dynamics with neurons firing bursts of spikes that vary substantially in timing and size across neurons (Supplementary Fig. 5b). In this regime,

the internal dynamics interact with incoming stimuli, creating a foundation for more complex information processing.

We examined how spiking irregularity of neurons changes across dynamical regimes as we increase the synaptic coupling strength J, driving the network from classical asynchronous activity to heterogeneous asynchronous activity. Although balanced excitatory-inhibitory networks are known to produce diverse and variable spiking activity, it has not been tested whether this variability arises from spiking irregularity or firing-rate fluctuations. To address this question, we applied our approach to partition total spiking variability into spiking irregularity ϕ and firing rate fluctuations across a range of synaptic strength values J.

We find that in the classical weak-coupling asynchronous regime (small J), neurons exhibit sub-Poisson spiking irregularity with narrow distribution of ϕ across neurons (Supplementary Fig. 5c). For larger J, the distribution broadens and the average ϕ increases, such that neurons exhibit super-Poisson irregularity with diverse ϕ in the strong-coupling regime (Supplementary Fig. 5c). Since each neuron in this model receives the same number of excitatory and inhibitory connections, the diverse spiking irregularity arises solely from recurrent dynamics in the heterogeneous asynchronous regime, providing a mechanism distinct from the heterogeneity in synaptic input balance (Fig. 6b-d). However, this random balanced network model generates sub-Poisson spiking irregularity only with narrow ϕ distribution, whereas the LIP and PMd data exhibit a broad range of sub-Poisson spiking irregularity across neurons (Fig. 4a). These results suggest that heterogeneity in incoming synaptic connections in random balanced networks may be necessary to produce the diverse sub-Poisson spiking irregularity, similar to our LIP and PMd data.

It is commonly assumed that neurons fire with Poisson-like irregularity in the classical asynchronous regime [10]. Contrary to this assumption, we found that neurons in this regime exhibit sub-Poisson spiking irregularity; for example, the average spiking irregularity is approximately 0.5 at $J = 0.2$. In addition, while previous studies reported that total spiking variability increases with synaptic strength J [10, 11], our partitioning framework enables us to determine whether this increase results from increased spiking irregularity or firing-rate variability. We found that both spiking irregularity and firing rate variability increase with synaptic strength (Supplementary Fig. 5c,d), indicating that stronger synaptic connections elevate spiking variability on both short and long timescales. Thus, our partitioning framework provides new insights into dynamics of balanced networks across dynamical regimes governed by the synaptic strength parameter.

1.7 Modulation of spiking irregularity and firing rate

We used the spatial balanced spiking network model [12] (Fig. 6) to further test whether input-driven changes in the operating regime of network dynamics can produce a modulation of the spiking irregularity. On average, an increase in the excitatory input to a neuron sets it closer to the firing threshold, leading to an increase in the firing rate and a decrease in ϕ. This condition arises, for example, when we elevate the baseline input current to the excitatory neurons (Supplementary Fig. 6a), and the reverse effect happens when we elevate the baseline input current to the inhibitory neurons (Supplementary Fig. 6b). This mechanism is consistent with the general trend that ϕ is inversely related to the mean firing rate (Fig. 6c,d).

In our PMd data, however, both ϕ and the mean firing rate increased during the decision epoch (Fig. 5f), thus, we tested additional mechanisms that could produce such modulations. Besides shifting the excitation-inhibition balance, an external input can also dynamically modulate the

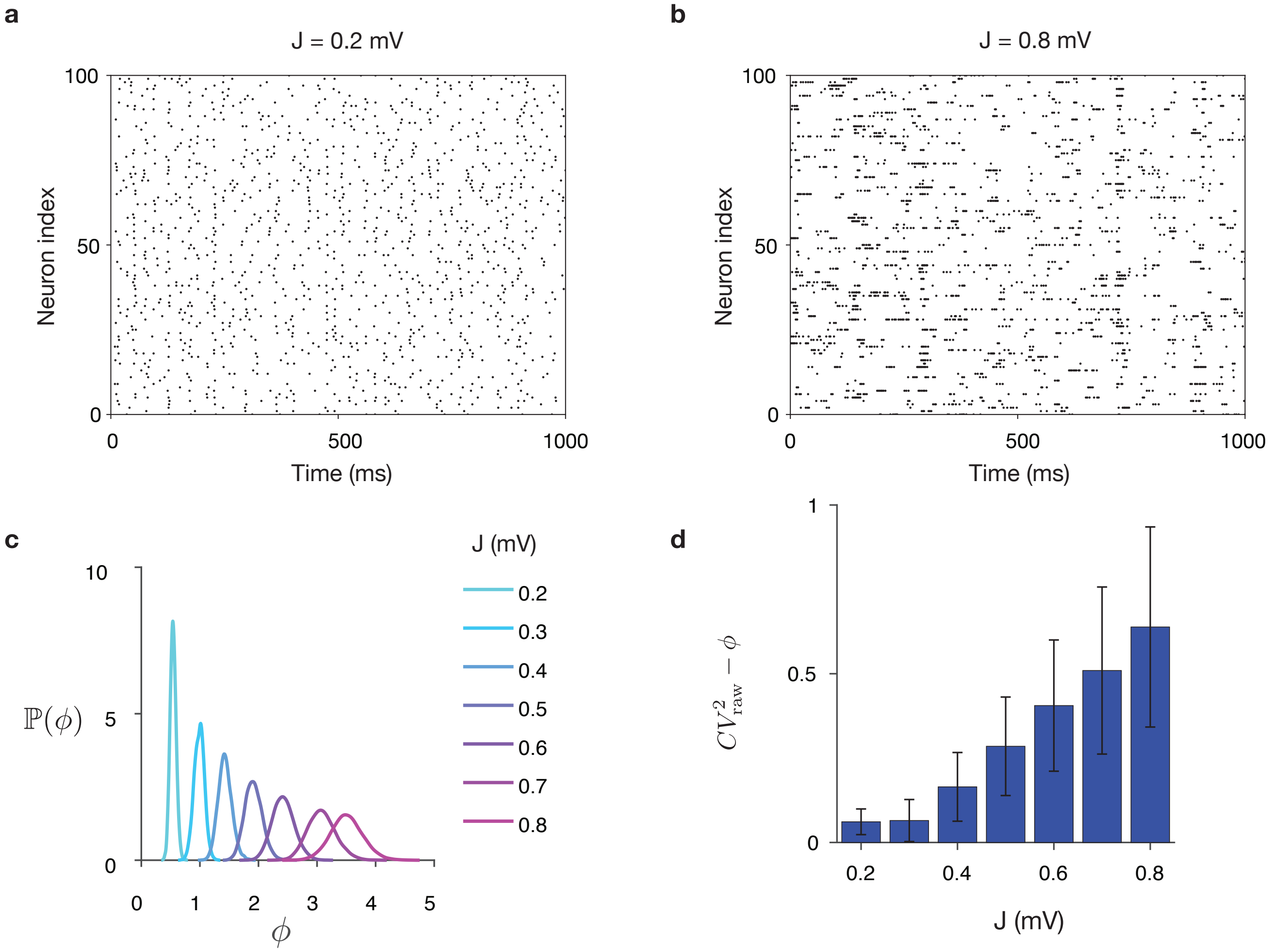

Supplementary Figure 5. Impact of dynamical regimes on spiking irregularity in the random balanced network model. **a**, In the classical asynchronous regime (weak synaptic coupling, $J = 0.2$ mv), the network exhibits asynchronous activity with similar irregular firing across all neurons. **b**, In the strong synaptic coupling regime ($J = 0.8$ mv), the network exhibits structured activity with neurons firing bursts of spikes that vary substantially in timing and size across neurons. **c**, Distribution of spiking irregularity ϕ across neurons for different values of the synaptic strength J. The average spiking irregularity increases with J. In the classical asynchronous regime (small J), the distribution of ϕ is narrow, whereas in the heterogeneous asynchronous regime (large J), the distribution of ϕ broadens indicating greater diversity in spiking irregularity across neurons. **d**, The difference between the total spiking variability (squared coefficient of variation CV^2_{raw} of ISIs in real time) and spiking irregularity ϕ increases with synaptic strength J, indicating that firing rate variability increases with J. The bar represents the mean value across 8,000 neurons, and the error bars indicate the standard deviation.

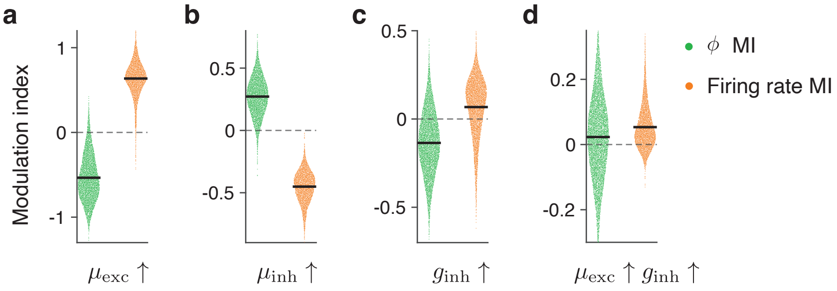

Supplementary Figure 6. Modulation of spiking irregularity and firing rate by input-driven changes in network dynamics. We analyzed how spiking irregularity ϕ (green) and mean firing rate (orange) change with the network state in Layer 2 of the spatially organized balanced spiking network model (Fig. 6). To simulate changes in the network state, we first set all parameters as specified in Tables 1, 2 (pre-state) and then modify only the parameters specified in each panel (post-state). Modulation index (MI) is defined as $2(\phi_{\mathrm{post}} - \phi_{\mathrm{pre}})/(\phi_{\mathrm{post}} + \phi_{\mathrm{pre}})$ for ϕ and analogously for the firing rate. **a**, Increasing the baseline input to the excitatory neurons μ_{exc} increases the firing rate and decreases the spiking irregularity ($p < 10^{-10}$, $n = 8,687$, two-sided t-test). The increased baseline input to excitatory neurons (μ_{exc} ↑) is modeled by changing μ_{exc} from 0 Vs^{-1} to 0.5 Vs^{-1}, with a membrane time constant $\tau_m = 10$ ms. **b**, Increasing the baseline input to the inhibitory neurons μ_{inh} has the opposite effect ($p < 10^{-10}$, $n = 7,094$, two-sided t-test). The increased baseline input to the inhibitory neurons (μ_{inh} ↑) is modeled by changing μ_{inh} from 0 Vs^{-1} to 0.5 Vs^{-1}, with a membrane time constant of excitatory neurons $\tau_m = 10$ ms. **c**, Decreasing the membrane conductance of the excitatory neurons g_{exc} increases the firing rate and decreases the spiking irregularity ($p < 10^{-10}$, $n = 9,002$, two-sided t-test). The decreased membrane conductance of the excitatory neurons (g_{exc} ↓) is modeled by changing τ_m for excitatory neurons from 11 ms to 20 ms. **d**, Increasing both μ_{exc} and g_{exc} increases both the firing rate and spiking irregularity ($p < 10^{-10}$, $n = 9,026$, two-sided t-test), as we observed in the experimental data for the decision versus fixation epoch (Fig. 5f). The modulation of the baseline input current and membrane conductance of the excitatory neurons (μ_{exc} ↑ g_{exc} ↑) is modeled by changing τ_m for excitatory neurons from 20 ms to 10 ms, and μ_{exc} from 0 Vs^{-1} to 0.02 Vs^{-1}. Dots represent individual neurons, horizontal lines indicate mean.

biophysical properties of a neuron, such as the membrane conductance [13, 14, 15]. In particular, a low-amplitude fast synaptic input simultaneously depolarizes a cell and increases its membrane conductance [16]. In the spatial balanced spiking network model, a reduction in the membrane conductance of excitatory neurons alone decreased the spiking irregularity while increasing the firing rate (Supplementary Fig. 6c). However, simultaneously depolarizing the excitatory neurons and increasing their membrane conductance increased both the spiking irregularity and firing rate (Supplementary Fig. 6d), similar to the modulation observed in PMd (Fig. 5f). This result shows that changes in spiking irregularity are not always inversely related to the firing rate. Therefore, multiple biophysical mechanisms can modulate spiking irregularity ϕ, and measurements of the spiking irregularity in experimental data can provide tighter constraints on biophysical neural circuit models.

1.8 Modeling serial ISI correlations with the DSR framework

While our DSR model assumes that interspike intervals (ISIs) are independent in operational time, it can generate serial ISI correlations in real time through temporally correlated instantaneous firing rate. Serial correlations between ISIs are observed in data[17, 18] and can arise from correlated input or firing-rate adaptation in mechanistic integrate-and-fire models[19, 20, 21, 22]. Thus, a question arises as to whether the DSR framework can capture serial ISI correlations resulting from correlated input or adaptation in mechanistic integrate-and-fire models. ISIs are approximately uncorrelated in real time in the low firing-rate regime, where the timescale of the correlated input or adaptation is much shorter than a typical ISI. Serial ISI correlations arise in the high firing-rate regime, where the timescale of the correlated input or adaptation is longer than a typical ISI. We therefore tested whether in this regime, our DSR model can capture serial ISI correlations through the slow dynamics of the instantaneous firing rate with approximately independent ISIs in operational time.

We used a leaky integrate-and-fire (LIF) neuron model driven by exponentially correlated Gaussian noise [20]. The dynamics of the membrane potential $V(t)$ are described by the equation

$$\tau_m \frac{dV(t)}{dt} = -V(t) + RI_s(t). \tag{16}$$

The neuron fires a spike when the membrane potential reaches a threshold V_{th}, after which the membrane potential is reset to V_r. The synaptic input current $I_s(t)$ is an Ornstein-Uhlenbeck process:

$$\tau_s \frac{dI_s(t)}{dt} = -I_s(t) + \mu + \sigma\xi(t), \tag{17}$$

with white Gaussian noise $\xi(t)$ that obeys $< \xi(t)\xi(t') >= \delta(t-t')$. We consider input with a long correlation time by setting $\tau = \tau_s/\tau_m = 200$ and $D = (\sigma^2 R^2)/(2\tau_m(V_{\mathrm{th}} - V_r)^2) = 0.3$. With these parameters, the neuron generates spikes showing strong serial ISI correlations (Pearson correlation coefficient between adjacent ISIs $r = 0.97$).

We tested whether our DSR framework can capture these serial ISI correlations using the same analysis that we developed for intracellular voltage recordings (Fig. 3). First, we estimated an empirical function relating the average subthreshold voltage to firing rate. Then, we use this function to compute the instantaneous firing rate from the subthreshold voltage. Finally, we use this estimated instantaneous firing rate to map spikes from real to operational time via

time rescaling. The ISI correlations in the operational time were considerably reduced (Pearson correlation coefficient between adjacent ISIs $r = 0.11$). This example illustrates that strong serial ISIs correlations in real time can be statistically captured by the DSR model using time-varying firing rate with approximately uncorrelated ISIs in operational time. Thus, the DSR framework is broadly applicable for modeling serial ISI correlations.

1.9 Demšar comparison test for spiking irregularity ϕ across task conditions

Condition	MR	MD	MAD	CI	Effect size	Magnitude
Cue-orth-2	2.316	1.153	0.398	[1.062, 1.270]	0.000	negligible
Cue-orth-1	2.422	1.144	0.424	[1.070, 1.274]	0.024	negligible
Cue-opp	2.570	1.150	0.375	[1.062, 1.275]	0.010	negligible
Cue-RF	2.692	1.129	0.404	[1.052, 1.245]	0.062	negligible

Supplementary Table 1. Demšar comparison test for ϕ between attention conditions in V4 data. MR - mean rank, MD - median, MAD - median absolute deviation, CI - confidence interval of MD, effect size - Cohen's effect size.

Comparison of ϕ across attention conditions in V4. We conducted the statistical analysis of the spiking irregularity ϕ for a population of 237 neurons in 4 attention conditions (c1: Cue-orth-2, c2: Cue-orth-1, c3: Cue-opp, c4: Cue-RF). The family-wise significance level of the tests is $\alpha = 0.05$. Based on the Shapiro-Wilk test, we rejected the null hypothesis that the population is normal for all conditions ($p < 10^{-4}$). Because we have more than two populations and all of them are not normal, we use the non-parametric Friedman test as an omnibus test to determine whether there are any significant differences between the median values of the populations. We use the post-hoc Nemenyi test to infer which differences are significant.

We report the median (MD), the median absolute deviation (MAD), and the mean rank (MR) among all populations over the samples (Supplementary Table 1). Differences between populations are significant if the difference in the mean rank is greater than the critical distance CD=0.305 of the Nemenyi test. Based on the post-hoc Nemenyi test, we conclude that there are no significant differences within c1, c3, and c4 ($p = 0.071$). We also conclude that there are no significant differences within c1, c2, and c4 ($p = 0.106$). If we consider all four conditions together they are significantly different ($p = 0.009$) with the maximum effect size of 0.062 (Supplementary Table 1). Since the effect size is smaller than the critical value of 0.2, it classifies as a negligible effect [23]. Therefore, we conclude that the differences in ϕ between the four attention conditions are negligible.

Comparison of ϕ between two-choice and four-choice decision-making tasks in LIP. We conducted the statistical analysis of the spiking irregularity ϕ for a population of 60 neurons during four-choice (c1) and two-choice (c2) decision-making tasks. The family-wise significance level of the tests is $\alpha = 0.05$. Based on the Shapiro-Wilk test, we rejected the null hypothesis that the population is normal for both populations ($p < 10^{-4}$). No check for homogeneity was required because we only have two populations. Because we have only two populations and both of them are not normal, we use the Wilcoxon signed rank test to determine the differences in

Task	MR	MD	MAD	CI	Effect size	Magnitude
4 choices	1.483	0.546	0.291	[0.448, 0.859]	0.00	negligible
2 choices	1.517	0.509	0.264	[0.450, 0.788]	0.14	negligible

Supplementary Table 2. Demšar comparison test for ϕ during the two-choice and four-choice decision-making tasks in the LIP data. MR - mean rank, MD - median, MAD - median absolute deviation, CI - confidence of MD interval, effect size - Cohen's effect size.

the central tendency and report the median (MD), the median absolute deviation (MAD), and the mean rank (MR) for each population (Supplementary Table 2). We failed to reject the null hypothesis ($p = 0.109$) of the Wilcoxon signed rank test that population c2 (MD$= 0.546 \pm 0.206$, MAD=0.291) is not greater than population c1 (MD$= 0.509 \pm 0.169$, MAD=0.264). Therefore, we conclude that there is no statistically significant difference in ϕ between two-choice and four-choice decision-making tasks.

Task epoch	MR	MD	MAD	CI	Effect size	Magnitude
Decision	1.422	0.453	0.180	[0.407, 0.735]	0.00	negligible
Fixation	1.578	0.497	0.249	[0.375, 0.753]	-0.204262	small

Supplementary Table 3. Demšar comparison test for ϕ in the decision and fixation epochs of the decision-making task in LIP. MR - mean rank, MD - median, MAD - median absolute deviation, CI - confidence interval of MD, effect size - Cohen's effect size.

Comparison of ϕ between decision and fixation epochs of the task in LIP. We conducted the statistical analysis of the spiking irregularity ϕ for a population of 45 neurons during the decision (c1) and fixation (c2) epochs of the decision-making task. The family-wise significance level of the tests is $\alpha = 0.05$. Based on the Shapiro-Wilk test, we rejected the null hypothesis that the population is normal for the populations c1 ($p < 10^{-4}$) and c2 ($p = 0.005$). No check for homogeneity was required because we only have two populations. Because we have only two populations and both of them are not normal, we use the Wilcoxon signed rank test to determine the differences in the central tendency and report the median (MD), the median absolute deviation (MAD), and the mean rank (MR) for each population (Supplementary Table 3). We failed to reject the null hypothesis ($p = 0.844$) of the Wilcoxon signed rank test that population c2 (MD$= 0.453 \pm 0.164$, MAD=0.180) is not greater than population c1 (MD$= 0.497 \pm 0.189$, MAD=0.249). Therefore, we conclude that there is no statistically significant difference in ϕ between decision and fixation epochs of the decision-making task in LIP.

Comparison of ϕ across task conditions in PMd. We conducted the statistical analysis of the spiking irregularity ϕ for a population of 272 neurons across 14 task conditions (c1 to c14, seven stimulus coherence levels for each left and right response side). The family-wise significance level of the tests is alpha=0.050. Based on the Shapiro-Wilk test, we rejected the null hypothesis that the population is normal for all 14 populations ($p < 10^{-4}$). Because we have more than two populations and all of them are not normal, we use the non-parametric Friedman test as an omnibus test to determine if there are any significant differences between the median values of the populations. We use the post-hoc Nemenyi test to infer which differences are significant. We

Task condition	MR	MD	MAD	CI	Effect size	Magnitude
c1	7.581	0.569	0.244	[0.483, 0.661]	-0.05	negligible
c2	7.618	0.579	0.252	[0.488, 0.673]	-0.08	negligible
c3	7.474	0.569	0.239	[0.499, 0.664]	-0.05	negligible
c4	7.346	0.576	0.278	[0.498, 0.667]	-0.07	negligible
c5	7.772	0.576	0.262	[0.488, 0.650]	-0.07	negligible
c6	7.305	0.558	0.238	[0.503, 0.641]	0.00	negligible
c7	7.820	0.555	0.235	[0.488, 0.652]	0.01	negligible
c8	8.033	0.550	0.258	[0.472, 0.623]	0.02	negligible
c9	7.846	0.555	0.259	[0.481, 0.641]	0.01	negligible
c10	7.522	0.570	0.260	[0.490, 0.654]	-0.05	negligible
c11	7.460	0.550	0.254	[0.491, 0.656]	0.03	negligible
c12	6.967	0.557	0.276	[0.492, 0.657]	0.00	negligible
c13	7.232	0.552	0.265	[0.504, 0.662]	-0.02	negligible
c14	7.026	0.574	0.244	[0.487, 0.670]	-0.06	negligible

Supplementary Table 4. Demšar comparison test of ϕ across task conditions in PMd. Task conditions (c1 to c14) correspond to seven stimulus coherence levels for each left and right response side. MR - mean rank, MD - median, MAD - median absolute deviation, CI - confidence interval of MD, and effect size - Cohen's effect size.

report the median (MD), the median absolute deviation (MAD), and the mean rank (MR) among all populations over the samples (Supplementary Table 4). Differences between populations are significant if the difference in the mean rank is greater than the critical distance CD=1.203 of the Nemenyi test. We failed to reject the null hypothesis ($p = 0.112$) of the Friedman test that there is no difference in the central tendency of the populations. Therefore, we conclude that there is no statistically significant difference between the median values of the populations.

Task epoch	MR	MD	MAD	CI	Effect size	Magnitude
Decision	1.710	0.461	0.183	[0.403, 0.543]	0.000	negligible
Fixation	1.290	0.530	0.159	[0.468, 0.616]	-0.271	small

Supplementary Table 5. Demšar comparison test for ϕ between the decision and fixation epochs of the decision-making task in PMd. MR - mean rank, MD - median, MAD: median absolute deviation, CI - confidence interval of MD, and effect size - Cohen's effect size.

Comparison of ϕ between decision and fixation epochs of the trial in PMd. We conducted the statistical analysis of the spiking irregularity ϕ for a population of 262 neurons during the decision (c1) and fixation (c2) epochs of the decision-making task. The family-wise significance level of the tests is alpha=0.050. Based on the Shapiro-Wilk test, we rejected the null hypothesis that the population is normal for the populations c1 ($p = 0.011$) and c2 ($p < 10^{-4}$). No check for homogeneity was required because we only have two populations. Because we have only two populations and both of them are not normal, we use the Wilcoxon's signed rank test to determine the differences in the central tendency and report the median (MD), the median

absolute deviation (MAD), and the mean rank (MR) for each population (Supplementary Table 5). We reject the null hypothesis ($p < 10^{-10}$) of the Wilcoxon's signed rank test that population c1 (MD= 0.461 ± 0.070, MAD=0.183) is not greater than population c2 (MD= 0.530 ± 0.074, MAD=0.159). Therefore, we conclude that the median of ϕ is significantly greater for c1 than c2 with an effect size of -0.271. This effect size is classified as a small effect size [23].

References

[1] Cox, D. R. *Renewal Theory* (Springer, 1967).

[2] Cox, D. R. & Lewis, P. A. *The Statistical Analysis Of Series Of Events* (Springer, 1966).

[3] Grimmett, G. & Stirzaker, D. *Probability and Random Processes* (Oxford University Press, 2020).

[4] Blitzstein, J. K. & Hwang, J. *Introduction To Probability* (Crc Press, 2019).

[5] Churchland, A. K. *et al.* Variance as a signature of neural computations during decision making. *Neuron* **69**, 818–831 (2011).

[6] Amarasingham, A., Geman, S. & Harrison, M. T. Ambiguity and nonidentifiability in the statistical analysis of neural codes. *Proc Natl Acad Sci USA* **112**, 6455–6460 (2015).

[7] Marcos, E. *et al.* Neural variability in premotor cortex is modulated by trial history and predicts behavioral performance. *Neuron* **78**, 249–255 (2013).

[8] Licata, A. M. *et al.* Posterior parietal cortex guides visual decisions in rats. *J Neurosci* **37**, 4954–4966 (2017).

[9] Sardi, S. *et al.* Long anisotropic absolute refractory periods with rapid rise times to reliable responsiveness. *Phys Rev E* **105**, 014401 (2022).

[10] Ostojic, S. Two types of asynchronous activity in networks of excitatory and inhibitory spiking neurons. *Nat Neurosci* **17**, 594–600 (2014).

[11] Brunel, N. Dynamics of sparsely connected networks of excitatory and inhibitory spiking neurons. *J Comput Neurosci* **8**, 183–208 (2000).

[12] Huang, C. *et al.* Circuit models of low-dimensional shared variability in cortical networks. *Neuron* **101**, 337–348 (2019).

[13] Destexhe, A., Rudolph, M., Fellous, J.-M. & Sejnowski, T. J. Fluctuating synaptic conductances recreate in vivo-like activity in neocortical neurons. *Neurosci* **107**, 13–24 (2001).

[14] Monier, C., Chavane, F., Baudot, P., Graham, L. J. & Frégnac, Y. Orientation and direction selectivity of synaptic inputs in visual cortical neurons: a diversity of combinations produces spike tuning. *Neuron* **37**, 663–680 (2003).

[15] Richardson, M. J. Effects of synaptic conductance on the voltage distribution and firing rate of spiking neurons. *Phys Rev E* **69**, 051918 (2004).

[16] Destexhe, A. & Paré, D. Impact of network activity on the integrative properties of neocortical pyramidal neurons in vivo. *J Neurophysiol* **81**, 1531–1547 (1999).

[17] Farkhooi, F., Strube-Bloss, M. F. & Nawrot, M. P. Serial correlation in neural spike trains: Experimental evidence, stochastic modeling, and single neuron variability. *Phys Rev E* **79**, 021905 (2009).

[18] Engel, T. A., Schimansky-Geier, L., Herz, A. V., Schreiber, S. & Erchova, I. Subthreshold membrane-potential resonances shape spike-train patterns in the entorhinal cortex. *J Neurophysiol* **100**, 1576–1589 (2008).

[19] Liu, Y.-H. & Wang, X.-J. Spike-frequency adaptation of a generalized leaky integrate-and-fire model neuron. *J Comput Neurosci* **10**, 25–45 (2001).

[20] Schwalger, T. & Schimansky-Geier, L. Interspike interval statistics of a leaky integrate-and-fire neuron driven by gaussian noise with large correlation times. *Phys Rev E* **77**, 031914 (2008).

[21] Bauermeister, C., Schwalger, T., Russell, D. F., Neiman, A. B. & Lindner, B. Characteristic effects of stochastic oscillatory forcing on neural firing: analytical theory and comparison to paddlefish electroreceptor data. *PLoS Comput Biol* **9**, e1003170 (2013).

[22] Ramlow, L. & Lindner, B. Interspike interval correlations in neuron models with adaptation and correlated noise. *PLoS Comput Biol* **17**, e1009261 (2021).

[23] Juandi, D. *et al.* The effectiveness of dynamic geometry software applications in learning mathematics: A meta-analysis study. *International Association of Online Engineering* (2021).
